# Leadership is the essential non-technical skill in the trauma team - results of a qualitative study

**DOI:** 10.1186/1757-7241-17-48

**Published:** 2009-09-26

**Authors:** Magnus Hjortdahl, Amund H Ringen, Anne-Cathrine Naess, Torben Wisborg

**Affiliations:** 1 Department of Acute Care, The BEST Foundation- Better & Systematic Trauma Care, Hammerfest Hospital, Hammerfest, Norway; 2 Department of Ambulance Service, Division of Prehospital medicine, Oslo University Hospital, Norway

## Abstract

**Background:**

Trauma is the leading cause of death for young people in Norway. Studies indicate that several of these deaths are avoidable if the patient receives correct initial treatment. The trauma team is responsible for initial hospital treatment of traumatized patients, and team members have previously reported that non-technical skills as communication, leadership and cooperation are the major challenges. Better team function could improve patient outcome. The aim of this study was to obtain a deeper understanding of which non-technical skills are important to members of the trauma team during initial examination and treatment of trauma patients.

**Methods:**

Twelve semi-structured interviews were conducted at four different hospitals of various sizes and with different trauma load. At each hospital a nurse, an anaesthesiologist and a team leader (surgeon) were interviewed. The conversations were transcribed and analyzed using systematic text condensation according to the principles of Giorgi's phenomenological analysis as modified by Malterud.

**Results and conclusion:**

Leadership was perceived as an essential component in trauma management. The ideal leader should be an experienced surgeon, have extensive knowledge of trauma care, communicate clearly and radiate confidence. Team leaders were reported to have little trauma experience, and the team leaders interviewed requested more guidance and supervision. The need for better training of trauma teams and especially team leaders requires further investigation and action.

## Introduction

Trauma is the leading cause of death in the first four decades of life in Norway [1]. Esposito and colleagues have indicated that one out of four deaths caused by trauma can be prevented with better trauma care [2], and found that the preventable death rate declined to 15% after systems improvement [3]. Chiara and colleagues found that 43% of deaths caused by trauma were possibly preventable. They also found that over 50% of trauma patients received inappropriate treatment in hospital [4]A quite recent study revealed that most treatment errors still occur in the emergency room phase, and found that one of 13 deaths was deemed potentially preventable [5].

The trauma team is a complex organisation which has to work smoothly in stressful situations. The number of team members and the condition of the traumatized patients create great challenges for the trauma team. In Norwegian hospitals the trauma teams do not have fixed members, thus members attending the team may vary from one situation to the next. This variation contributes to the many challenges in team interaction. There is also a significant variation between the hospitals in terms of trauma load and thus experience in handling traumatized patients. Hospitals vary in size from small hospitals with few traumatized patients to hospitals with up to 3 traumatized patients daily. Different programs have been created to educate trauma team members for such situations. The BEST Foundation: Better & systematic trauma care developed a Norwegian training model using simulations for team training of hospital trauma teams. The focus for this training program is on non-technical skills as communication, leadership and cooperation. We have previously studied the effect of this training and also pointed out the greatest challenges for teamwork [6,7]. The result suggests that lack of communication, cooperation and leadership were the main obstacles experienced by the team members during their last real trauma situation before the team-training program. These results were obtained in written questionnaires with limited response alternatives. We believe that to optimize training, it is important to get more knowledge concerning training goals, and thus to elaborate on the need for non-technical skills.

The aim of this study was to obtain a deeper understanding of which non-technical skills are important in trauma teams when treating trauma patients. Topics such as cooperation, communication, education and leadership were included in an interview-guide used to elaborate the trauma team experiences.

Non-technical skills can be defined as behaviours in the operating room environment not directly related to the use of medical expertise, drugs or equipment. They encompass both interpersonal skills e.g. communication, teamwork, leadership, and cognitive skills e.g. situation awareness and decision making. [8] Leadership can be defined as the process of influencing the activities of an individual or a group in efforts of goal accomplishment[9].

## Methods

### Approval

The study was approved by Norwegian Social Science Data Services (ref. 15820/08/12-2006). The Regional Committee for Medical Research (Health Region East) did not consider any need for approval given the nature of the study (e-mail dated Ida Nyquist, 17/10-2006).

### Participants

The sampling strategy aimed at talking to team members of different professions, with a variety in trauma knowledge, and with teams from different sized hospitals. Four hospitals were recruited; a small hospital with low trauma load, a medium sized hospital with medium trauma load, large hospital with low trauma load and a large hospital with high trauma load. This was done to get the most diversity in clinical trauma experiences, thus creating a broader picture. At each hospital one nurse, one team leader and one anaesthesiologist were interviewed individually. All team leaders were consultants or residents in the Department of Surgery. They all had to be involved in the initial trauma treatment. Demographic data about the interviewed trauma team members and their hospitals are given in table 1.

**Table 1:** Hospital size and the informants' professional experience

Large hospital with low trauma load	Experienced Nurse:
	Age: 30-40
Size: 333 beds	Years in profession:5
	
	Experienced Anaesthesiologist:
	Age: 40-50
	Years in profession:13
	
	Experienced Team leader:
	Age: 40-50
	Years in profession:5
	
Medium sized hospital with medium trauma load	Experienced Nurse:
	Age: 40-50
Size: 188 beds	Years in profession:27
	
	Experienced Anaesthesiologist:
	Age: 40-50
	Years in profession:17
	
	Inexperienced Team leader:
	Age: 20-30
	Years in profession: 1
	
Small hospital with low trauma load	Experienced Nurse:
	Age: 20-30
Size: 46 beds	Years in profession: 4
	
	Experienced Anaesthesiologist:
	Age: 50-60
	Years in profession: 27
	
	Inexperienced Team leader:
	Age: 20-30
	Years in profession: 2
	
Large hospital with high trauma load	Experienced Nurse:
	Age: 30-40
Size: 726 beds	Years in profession: 11
	
	Experienced Anaesthesiologist:
	Age: 30-40
	Years in profession: 4
	
	Experienced Team leader:
	Age: 40-50
	Years in profession: 6

### Data collection

We conducted 12 semi-structured interviews which were tape recorded. Two of the authors participated in all 12 interviews (MH, AHR). An interview guide based on experiences from attending several trauma courses and observing trauma teams in action were used. The interview guide was discussed and adjusted after each interview. The Interview guide is presented in appendix 1. Written informed consent was obtained and information about the study was given at the start of the interview.

### Analysis

All 12 interviews were transcribed verbatim. The transcribed data were read through several times and a coding frame for the analysis was developed. Experiences concerning human factors in the trauma team were identified and used for systematic text condensation, according to the principles of Giorgi's phenomenological analysis modified by Malterud [10]. The analysis followed 4 steps described by Frich [11]: a) Reading all the material to get an overall impression, b) identifying units of meaning representing different experiences, and coding of these units, c) condensing and summarizing the contents of the coded groups and d) generalizing descriptions and concepts.

To support a valid interpretation of the data we frequently read the interviews again during the analysis. All quotes were plotted in a matrix to assess whether they were representative of a trend among the informants.

## Results

Leadership appeared to be the main determinant of team function during trauma team interaction. This finding was mutual between all informants. We grouped their descriptions of leadership in three categories: the successful leader, the solitary leader and the supportive team. The informants make it clear how important the leader was to them.

And most important of all is to have a defined leader who acts as a leader!

Experienced anaesthesiologist, large hospital with high trauma load

-I need to be comfortable with the leader and convinced that he solves his duties as a leader and that I recognize that the other team members carry out their responsibilities. First of all the leader is important. If he performs well, the team performs well.

Experienced anaesthesiologist, large hospital with low trauma load

Insufficient leadership was also pointed out as the reason for trauma management that failed.

### The successful leader

Professional competence was a quality that many team members appreciated in a leader. The team leader had to be trustworthy to the team members. The informants emphasized that the leader must have a special interest in emergency medicine. It was also pointed out that good leadership skills can not compensate for lack of trauma care knowledge. Several informants mentioned that a leader with high professional competence makes the team members confident. Some of the team members had bad experiences with inexperienced leaders.

The ability to radiate confidence and calmness was highlighted by several team members. One nurse said that if the leader seems confident she feels confident too. A young surgeon recalled a situation where he experienced a leader who remained calm in a stressful situation and emphasized that this had made a big impression on him.

One informant described a situation where an ambulance delivered a patient suffering from a ruptured abdominal aortic aneurysm.

They (the surgeons) did their job, but there was no affection in the situation at all. It was extremely effective (...). For me that was a milestone. To see how experienced surgeons handle a very, very serious situation. And I thought if it is possible in this context to remain calm, it will always be possible to act normal in an urgent situation.

Inexperienced surgeon, small hospital with low trauma load

An experienced team leader said that during the treatment of traumatized patients all the team members are alert and therefore it is his job to remain calm.

Everyone is more alert [during trauma treatment] and it is my responsibility to remain calm. I think that to have a functional team, you need a team leader who is calm and not stressed. If you are "stressed" you will make people around you stressed, this creates insecurity.

Experienced team leader, large hospital with high trauma load

The informants appreciated leaders that communicate distinctly and clearly. There should not be any room for misunderstandings about what the leader means and what he wants the team members to perform. At the same time the team leader should listen to and trust his team.

This is a description of a trauma team situation that failed:

The team leader was not distinct. It was not clear what findings he had and which decisions he made. When someone has to ask: What is the result of the investigation? What do you consider? Should we operate? In that situation you have to squeeze information out of the leader instead of him being clear with his decisions and considerations.

Experienced anaesthesiologist, medium sized hospital with medium trauma load

Most of the informants emphasised that a good leader needs to have an overview and see the totality of the situation. He has to help the process move forward and intervene if the process is going in the wrong direction. He needs to take responsibility and make decisions. The team members expect that the leader remain focused on what is important. Indecisive leaders were mentioned as the reason for unsuccessful trauma situations. An example was mentioned where the leader did not guide the team. The team leader became focused on single procedures and not the overall wellbeing of the patient.

That [a good leader] is a person who by his presence - makes the process evolve - not by his own efforts, but through guiding the team where it is needed - and intervening when necessary.

Experienced nurse, small hospital with low trauma load

### The solitary leader

Inexperienced team leaders often seemed in doubt whether or not they had the professional competence required for the given situation. They were also anxious about missing out on hidden, but serious injuries or indicated interventions. They explained that they did not get any experience in such decision making during their studies or internship. One team leader believed that there should always be an experienced surgeon in the Emergency department (ED), but admitted that this is not the case, and felt that the public should be made aware of this.

What makes you feel nervous about your position as a team leader?

If the trauma comes in at night, because then I am alone.

- Inexperienced team leader, medium sized hospital with medium trauma load

Inexperienced team leaders mentioned that it is a problem for them that they do not have experienced senior consultants present at night time. One resident mentioned his nervousness about performing a laparotomy at night time because his consultant was half an hour away from the hospital. The absence of consultants is one of the reasons why residents find trauma surgery more stressful than elective surgery. They feel more alone in the trauma setting.

-You are more alone in a trauma setting!

-What makes you feel secure in a trauma situation?

-That's easy to answer! To have more experienced people than me in the team. A good anesthesiologist, confident nurses that know where the equipment is - that's most important to me.

- Inexperienced resident surgeon, small hospital with low trauma load

Team members other than team leaders considered the leader's competence the most important factor determining their confidence in a trauma setting. They became insecure if the surgeon was newly educated or if the team leader was an experienced surgeon with little knowledge about trauma surgery.

A confident surgeon can easily get transformed into an insecure team leader. An urologist placed in the position as a team leader; in worst case scenario he has no competence in trauma care.

Experienced nurse, large hospital with low trauma load

There are no required qualifications for trauma team leaders in Norway. An experienced surgeon found this unfair both to the patients and the team leaders. *"A surgeon who has never even inserted a thoracic drain can suddenly find himself in the position as leader of a trauma team"*.

### The supportive team

All team members mentioned the importance of maintaining the authority of the leader in a situation where the leader needs help from the others. It was emphasized that the team needs to strengthen the leader, thus feedback to him needs to be constructive and given with respect. Team members therefore prefer to formulate feedback as questions or proposals like. "What do you think about his blood pressure and pulse"; "Should we take the patient to operating theatre?" It was important to the team members not to make the leader loose his face. This would make it impossible for the leader to fulfill his role as the leader following a conflict. Team members reported that if they direct the leader in the wrong way they could destroy his confidence and the team's trust in him. One nurse described the difficulty in correcting the leaders, even when their decisions were clearly wrong.

If the anesthesiologist does it (direct the leader) in a positive way and is more educating than self promoting, it usually turns out all right. It should not be a problem at all. On the other hand, if the anesthesiologist has an arrogant attitude, he can destroy the leader completely.

Experienced nurse, medium hospital with medium trauma load

The team leaders reported always to be open to criticism from the team. However, they wanted the feedback distinct and clear. An experienced surgeon told us that he is always ready for suggestions, but he was not always ready to discuss the suggestions in the trauma room. He emphasized the need for a clear command line. *"We have a command line, and that has to be respected by the rest of the team (...) In a team with an unstructured command line, the team members don't know who they should listen to, and they'll get confused"*. To an inexperienced surgeon, good leadership means listening to more experienced team members. Team leaders expected that the rest of the team would guide them if they were about to carry out wrong decisions. Anyhow, it came clear that too much discussion in the trauma room can make decisions more difficult to make.

Less important details can wait, but if team members have suggestions that can affect the immediate future, I expect them to speak out!

Inexperienced surgeon, small hospital with low trauma load

Some team members suggested that if the situation becomes too dysfunctional, a new leader should be appointed. Other members remember change of leadership as a bad experience. Others again had experienced team members who did not give the leader the opportunity to perform his role as a leader resulting in insecurity on the leader's behalf.

-Have you ever experienced that someone has taken over the leader's position? And did this create a pronounced change in the team structure?

-It has never been explicit, but it has happened anyway. It doesn't promote effective teamwork, to tell the truth. It is not fair to the leader, and it creates insecurity in the team when it comes to interaction and communication. Who are you going to report to in that situation?

Experienced nurse, big hospital with great trauma load

## Discussion

This study aimed at assessing which non-technical skills are important in the trauma team during trauma patient treatment. Several of our informants reported that leadership was one of the most important factors in appropriate trauma treatment. Lack of leadership was often given as a reason for dysfunctional teamwork. Recent research has confirmed this suggestion that the team leader has a major impact on the trauma team performance, and thereby ultimately much of the responsibility for the team's success or failure [12].

We were surprised to see that when asked how to be good at non-technical skills like leadership, many of our informants emphasised the importance of technical skills. One could not be a good team leader without being a skilled trauma surgeon. It seems like that to our informants, technical and non-technical skills are closely linked, and dependent of each other. The distinction between non-technical skills and experience in the technical trauma care related to operative and treatment experience was not distinct among the informants. Professional technical competence seems to radiate confidence in team members, and one can speculate whether inexperienced surgeons with good non-technical skills are met with less confidence despite their ability to fulfil the non-technical expectations of team members.

We found that our informants' thoughts concerning successful leadership were much the same as the NOTTS (Non-Technical Skills for Surgeons) taxonomy identified by University of Aberdeen Industrial Psychology Research Centre. This taxonomy is being used in the training of surgeons in non-technical skills by The Royal College of Surgeons of Edinburgh[13]. In Table 2 we have framed our findings using the NOTTS taxonomy.

**Table 2:** Findings grouped after the NOTTS (Non-Technical Skills for Surgeons) (13) taxonomy

**Category**	**Element**	**Our findings**
Situation awareness	Gathering information Understanding information Projecting and anticipating future state	Most of the informants emphasised that a good leader needs to have an overview and see the totality
Decision Making	Considering options Selecting and communicating option sImplementing and reviewing decisions	The leader needs to take responsibility and make decisions. There should not be any room for misunderstandings about what the leader means and what he/she wants the team members to perform.
Task Management	Planning and preparation Flexibility/responding to change	The leader has to help the process move forward and intervene if the process is going in the wrong direction.
Leadership	Setting and maintaining standards Supporting others Coping with pressure	The ability to radiate confidence and calmness was highlighted by several members. At the same time the team leader should listen to and trust his team.
Communication and Teamwork	Exchanging information Establishing a shared understanding Co-ordinating team activities	The informants appreciated leaders that communicate distinctly and clearly

Major expectations seem to be resting on the leader. It is possible that the team members exaggerate the leader's importance. Some of the expectations to the leader are not realistic. No leader can be the perfect communicator all the time. Communication without any misunderstanding is a great challenge. One could speculate if a confident team might compensate for a weak leader. If there is an experienced anesthesiologist in the trauma room it should be possible to share this responsibility. An editorial in the Journal of Trauma underlines the importance of the subordinate's role in making their leader good: *"Everyone has their blind spots!" *[14].

AC Edmondson describes how leaders can make a better environment for feedback to the leader. She has analyzed the process of promoting learning within interdisciplinary Action Teams(IATs) " In context in which formal power differences are present and speaking up matters for performance, it is incumbent upon those with power to find ways to minimize its silencing effect" Team leader coaching increase ease of speaking up in IATs and coaching will promote boundary spanning. Boundary spanning is important to make team members taking the risk of speaking up across team boundaries. [15]

One study conserning leadership of resuscitation teams found no direct coherence between ALS training and enhanced leadership. It was, however, found that better trained leaders did practice leadership with less time hands on, and that leaders with less time hands on would promote better teamwork. The quality of the leadership did improve with the number of resuscitation attempts the leader has participated in. [16].

Knowledge seems to make team leaders confident. Therefore it is not surprising that this is mentioned as important by several team members. Professional competence gives the leader authority. It will also give the leader a sense of confidence that might make it easier to live up to the expectations resting upon him. A study of pediatric residents showed that improving technical skills made the residents more confident in their leadership [17]. A confident leader with little medical competence is even more dangerous to the team work and for the patient's wellbeing.

Several informants mentioned that the team should guide the team leader by focusing on the patient and not through direct criticisms. In this way, the leader can maintain his authority. Inadequate communication can make these discussions evolve to dysfunctional cooperation between different professions, as indicated in a study from London 2006 [12]. A textbook of leadership function and training illustrates inadequate communication/dysfunctional cooperation with a study where 37 air plane accidents were analyzed; in 31 of these one crew member failed to detect and challenge another crew member's error, usually the captain's [18]. It seems like several of the interviewed team members think that guidance can ruin the leader's authority. A subtle way to help this is to focus on the patient without giving outspoken corrections. This can be in contradiction to the need of distinct communication.

Several of the inexperienced team leaders mentioned that they felt anxious when they were the sole surgeon in the emergency room. There are great expectations to the leader and through the interviews it seems like not all residents feel prepared for this task. This burden of expectation is described in a Canadian study where 49% of the internal medicine residents felt inadequately trained to lead a cardiac arrest team [19].

## Validity and Transferability

The initial aim of our study was to unveil which non-technical skills trauma team members considered important in the trauma team when treating trauma patients. During the process of interviewing it turned out that leadership was a major determinant to all informants. We therefore decided to omit a number of other findings on the subjects such as communication, team work and training. This is in accordance with the method applied [10].

This study explores the experiences of team members working in a trauma team. Talking about conflicts and co-workers may be uncomfortable to the informants. As the interviewers were medical students the challenge of this was probably less compared to being interviewed by colleagues. It might have been tempting to team members to ascribe all team difficulties to the team leader. The fact that also team leaders underlined leadership as a crucial factor, and acknowledged their own insufficiency, suggests a high level of openness and willingness to disclose also their unpleasant knowledge.

The sampling strategy allowed us to interview personnel with varying experience at different hospitals with varying trauma load. Therefore we think that our results are transferable to other trauma teams independent of hospital size. Our findings are supported by similar findings in two recent studies [12,19]. Hayes et al pointed out the problems with inexperienced team-leaders in stressful situations, and Cole & Crichton described challenges in teamwork and leadership in trauma management.

## Implications

Norwegian trauma-patients will be met by trauma team members that find experienced leaders as one of the key factors to successful trauma treatment. The team might still be led by a resident who seeks experience in the team around him. It seems necessary to explore the needs for training and education of team leaders. Better qualified and more confident team leaders might enhance the teams' performance. This should be confirmed by further studies.

## Competing interests

Travel expenses to perform the interviews and some of the transcription were covered by the BEST-network.

## Authors' contributions

MH and AHR did the data collection, analysis and the first draft writing. TW and CAN read through all the data and supervised the analysis and writing. TW conceived the study.

## Appendix 1

### Interview guide

General information:

Thank you for participating in our study.

We want to find out how the Norwegian trauma teams work and what the team members find important for the team to function. We will interview several team members and ask what they think is important. After that we will analyze this material and find the essence of the opinions. We want to publish these findings in a paper in a medical journal.

The interview will take about one hour. One of us will ask the questions. We will record the conversation. The interview will be anonymous and we encourage you to not use names, but refer to your colleges with the name of their roles in the trauma team. We will not focus on technical procedures.

### Part 1

1. Age: Specialty: Years with experience:

2. How do you find the trauma load at this hospital?

Low, medium, high

3. How often do you participate in the trauma team?

4. How do you find the trauma part of your responsibility at this hospital?

5. Please describe the composition of the trauma team at this hospital?

### Part 2

#### Teamwork

What do you think is most important for the team to work well together?

Please describe your experiences of the team working together?

Please describe the cooperation between the different specialties?

### Leadership

What is a good team leader to you?

Please describe your experiences of leadership of the trauma team?

What education for the role as a leader have you received?

How has med. school prepare you for that role?

### Communication

What is good communication to you?

Please describe your experiences of the communication in the team?

In situations where the teamwork works well, how is the communication in those cases?

And in situations that it does not work?

### Education

How are you prepared for working in the trauma team?

Do you get enough education for your tasks in the trauma team?

How did medical school prepare you for these kinds of challenges?

### General

What makes you confident in a trauma setting?

If you look back at your last trauma situation, what made that situation successful/unsuccessful?

Do you have any suggestions for changes of the trauma team?

What make you insecure if you have trauma call?

Do you have anything to add at the end of the interview?
